# Using the Health Belief Model to Explain the Patient's Compliance to Anti-hypertensive Treatment in Three District Hospitals - Dar Es Salaam, Tanzania: A Cross Section Study

**DOI:** 10.24248/eahrj.v5i1.651

**Published:** 2021-06-11

**Authors:** Angelina Alphonce Joho

**Affiliations:** a School of Nursing and Public Health, Department of Clinical Nursing, University of Dodoma, Tanzania

## Abstract

**Background:**

Hypertension remains a public-health challenge globally. Its prevention, early detection, proper and adequate treatment and control should be given high consideration to prevent occurrence of cardiovascular disease and stroke. This study is guided by the Health Belief Model (HBM) to investigate the influence of treatment compliance using HBM constructs among elderly hypertensive patients in 3 regional hospitals in Dar es Salaam, Tanzania.

**Methods:**

An analytical cross-sectional study was conducted in 3 region hospitals in Dar es Salaam from April to May 2012. The study included patients who were on antihypertensive medications. Simple Random Sampling was used to enrol the study participants. Data was collected using structured questionnaire. Data was analysed using SPSS version 20. Linear Multiple Regression analysis was performed to identify variables which are strongest predictor of treatment compliance among variables of the Health belief Model.

**Results:**

A total of 135 participants were enrolled of whom 56% were compliant to hypertensive treatment. Multivariate analysis indicated significant model fit for the data (F=11.19 and *P value* <*.001*). The amount of variance in treatment compliance that was explained by the predictors was 30.3% (R^2^=0.303) with perceived barrier being the strongest predictor of treatment compliance (β=−0.477; *p*< *.001*). Other predictor variables were not statistically associated with treatment compliance.

**Conclusion:**

The study showed that 56% of study participants had hypertensive treatment compliance and perceived barrier to treatment was the strongest predictor. Innovative strategy on improving patients’ perception of barrier to treatment is recommended in order to improve treatment compliance.

## BACKGROUND

Hypertension remains a public-health challenge globally. It is the main modifiable independent risk factor for development of cardiovascular disease, stroke and renal failure which increase significantly with age^1^. Prevention, early detection, proper and adequate treatment and control should be given high consideration to prevent occurrence of cardiovascular disease and stroke.^2^ Control and prevention of hypertension complications can only happen when individuals recognise the benefits of changing lifestyle behaviour and believe that they are susceptible to hypertension complications.^3^

Health Belief Model (HBM) has been used to explain and predict individuals health behaviours for preventing and/or controlling diseases and their complications by including perception of susceptibility, seriousness, severity, benefits and barriers to a health behaviour and cues to action.^3^ The global burden of hypertension projects indicate that the number of adults with hypertension will increase by 60% to a total of 1.56 billion in 2025.^4^ For Sub-Saharan Africa, the prevalence of hypertension has been projected to be 125.5 million among adults in 2025.^5^

In Tanzania, hypertension related diseases are the second cause of hospital admission and cause of deaths.^6^

Tanzania particularly in the cities like Dar es Salaam, people are experiencing urbanisation and modernisation. This has brought about changes in their lifestyle especially their diet intake and physical activity. This has led to overweight, obesity and physical inactivity which altogether increase the risk for hypertension and cardiovascular diseases.^7,8^

Management of hypertension requires medication and lifestyle compliance. The lifestyle modification includes; increase in exercise, lowering of body mass index, reduced-sodium diet, moderation of alcohol consumption and quitting smoking.^9^ These lifestyle modifications and taking medication properly are examples of therapeutic behaviours.^9^ The treatment guideline of hypertension in Tanzania are either nonpharmacological or pharmacological treatment. Nonpharmacological treatment calls for life style modification which includes weight reduction, adopting dietary approaches to stop hypertension (DASH) such as eating diet rich in fibre-fruits, vegetable, unrefined carbohydrate and low-fat dairy products with reduced content of saturated and total fat. Also, reduction in dietary sodium intake, involvement in regular physical activity such as a brisk walking for at least 30 min/day 3 days a week, stop the use of all tobacco products and reducing alcohol consumption.^10^ Pharmacological treatment includes combination of drugs including diuretics, Angiotensin-Converting Enzyme Inhibitor (ACEI), Angiotensin Receptor Blocker-ARB, Beta-blocker and Calcium Channel Blocker.^10^

Uncontrolled hypertension is caused mainly by non-adherence to the antihypertensive drugs^11^ and lifestyle.^12^ Understanding drug regimens by the patients helps to improve their adherence, and thus preventing the complications related to hypertension which are debilitating and if not prevented may increase the burden of cardiovascular diseases.^12^ Adhering to antihypertensive drugs remains to be an important modifiable factor towards management of hypertension. Non adherence to anti-hypertensive agents seriously affect the effectiveness of treatment and thus causing an increase in cardiovascular and cerebro vascular risks and consequently causing population health problems in the quality of life as well as health economics. Non-adherence to pharmaceutical therapy is a major problem all over the world. Studies on drug adherence to chronic diseases such as hypertension show that adherence is about 67.2%.^11^

HBM is an approach that is used to describe social behaviour as well as individual's cognition. It was introduced in the 1950s by Social psychologists so as to facilitate in reasoning individual's participation in health programs such as health check-ups and immunisation.^14^ The HBM was also widely used to explain a range of health behaviour. The model also base on studying compliances with lifestyle modification and antihypertensive medication, including understanding that high blood pressure involves both drug treatment and lifestyle changes.

**FIGURE 1:**
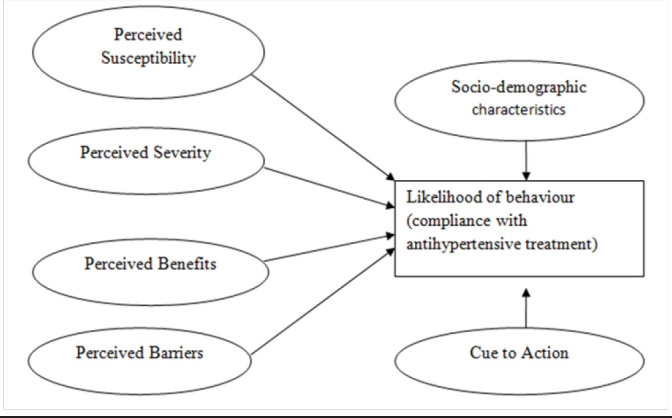
Inter-relationship between Variables of Health Belief Model which were used to explain Hypertension Treatment Compliance

According to Rosenstock et al, the HBM constructs reported were perceived susceptibility, perceived severity, perceived barriers, perceived benefits and cues to action hypothesises that health-related action depends upon an individual's motivation, belief of being vulnerable to a disease and one's belief to certain health recommendations that is important in befitting health and reduce disease complications.^15^ (Figure 1).

Little is documented on the influence of health belief model variable on compliance to hypertensive treatments. Therefore, this study aimed at assessing the influence of Health Belief Model variable on hypertensive treatment compliance among hypertension patients in 3region Hospitals in Dar es Salaam, Tanzania.

## METHODS

### Study Design and Setting

We conducted an analytical cross-sectional study design from April to May 2012 using quantitative approach. The study included data from 3 regional hospitals in Dar es Salaam; Amana, Mwananyamala and Temeke hospitals which are located in Ilala, Kinondoni and Temeke Municipalities respectively. The estimated population in these municipalities is 1,220,611, 1,775,049 and 1,368,881 people for Ilala, Kinondoni and Temeke Municipalities respectively.^16^ The study sites were selected because majority of hypertensive patients who are diagnosed from primary health facilities are referred to those hospitals for expert management.

### Study Population

The study population included patients with hypertension who were using antihypertensive treatment and were attending hypertension clinics from the selected hospitals. Each Hospital has 2 days per week of clinic for non-communicable diseases including hypertension. All 3 hospitals have inpatient and outpatient services.

### Sample Size and Sampling Procedure Sample Size

Sample size of 135 participants was calculated using kish and Leslie formula (1965).

$$\text{n}={\text{Z}}^{2}\text{p}(1-\text{p})/{\text{e}}^{2}.$$

The prevalence used was 34% compliance in Temeke-Dar es Salaam.^17^

Where: n = the required minimum sample size

ε = margin of error (5%)

p = estimated proportion of compliance 34%

z = standard normal deviate corresponding to 95% confidence level=1.96

Considering a margin error of 5% and a 95% confidence level, the minimum required sample size obtained was 135.

### Sampling Procedure

The 3 regional hospitals were selected purposively because most of the patient who have hypertension and hypertensive complications are referred to these hospitals from the Primary Health Facilities. We reviewed the records of hypertensive patients attending in each of the 3 hospitals for the period of 1 month. We found that 366, 583 and 340 patients attended Amana hospital, Mwanayamala hospital, and Temeka hospital respectively. The 135-sample size calculated was proportionally allocated to 3 hospitals based on the above records. Thus, 39 participants were selected from Amana, 60 from Mwananyamala and 36 from Temeke hospital. Simple random sampling was used to select the study participants. The researcher established a sampling list from patients attending hypertensive clinics to the respective study hospitals to obtain eligible participants that meet the inclusion criteria. There were pieces of paper that were written on; YES or NO. The word “YES” was used to represent the targeted study population, and “NO” was used to represent the population that was not going to participate in the study. The procedure of drawing papers from the box by each study participant was used. Once apiece of paper was picked, it was not included in the sample again and each participant was allowed to pick only once.

### The Inclusion Criteria and Exclusion Criteria

All patients aged 18 years and above with a diagnosis of hypertension for at least one month with or without other co-existing medical conditions and consented to participate in the study. Participants who had been taking anti-hypertensive treatment for at least one month before the beginning of the study were included. Allpatients who had not started antihypertensive treatment and could not respond because of reasons such as being too sick to be interviewed were excluded from the study.

### Measures of Outcome

The outcome variable was treatment compliance which comprised of medication regimen compliance and lifestyle modification. Medication regimen compliance was composed of 8 items, asking “how often you forget to take your medicine”. The responses were measured on a 4-point Likert scale: “1” Every day, “2” frequently, “3” rarely or “4” never. For life style, compliance was having 5 items, participants were asked to respond to the single question based on a 4-point Likert scale: “How often do desirable or undesirable behaviours related to control of hypertension”. The responses were: “1” Every day, “2” frequently, “3” rarely or “4” never. Some questions were set such that the highest score did not reflect the worst scenario of non-compliance. To resolve these, scores were reversed. For example, how often do you engage in physical exercise “4”, every day, “3” frequently, “2” rarely or “1” never. The 13 items measuring treatment compliance and life style compliance were added up to get sum index with a distribution ranging from 33 to 52 with mean 44.2963 (SD =3.32578), the median split was used (44.4), which was dichotomised into two groups i.e. 0 = those who are non-treatment compliant and 1 = treatment compliant which was 34–43 and 44–51.

The HBM constructs included perceived severity of having hypertension, perceived susceptibility of being at risk of hypertension complications and perceived benefit were each was measured by 6 items. The reminders (cues to action) were measured by 7 items. Participants were then asked to respond: “1” strongly agree, “2” agree, “3” disagree or “4” strongly disagree.

6 items measuring perceived severity were added up to get sum index with a distribution ranging from 7 to 24 with mean 20.10 (SD =2.85). The median split 50.4 was used as a cut-off point. Dichotomisation was done into 2 frequency groups; those who had low perceived severity and those who had high perceived severity. 6 items measuring perceived susceptibility were added up to get sum index ranging from 6 to 19 with mean of 10.79 (SD =2.83). The median split was 49.6. The sum index for perceived susceptibility was dichotomised into 1= those with low perceived susceptibility and 2= those with high perceived susceptibility.

6 items measuring perceived benefit were added up to get sum index with a distribution ranging from 12 to 24 with mean (SD) 20.24(2.87) and median split 51.1. Then dichotomised into; those with low perceived benefit and those with high perceived benefit. 7 items measuring cues to action were added up to get sum index with a distribution ranging from 15 to 28 with mean (SD) 24.27(2.65) median split 42.2, then dichotomised into 2 frequency groups; those with low perceived cues to action and those with high cues to action.

5 items measuring perceived barriers were added up to get sum index with a distribution ranging from 5 to 15 with mean (SD) 8.36 (2.48), median split 54.8, then dichotomised into; those with low perceived barrier and those with high perceived barrier. The aspects that might hinder respondents to comply with their treatment included not having enough time to do exercise. Responses were “1” strongly not a problem, “2” not a problem, “3” problem and “4” strongly a problem.

Bivariate analysis using Chi-squire and Pearson correlation between HBM variables were performed. Multiple linear regression analysis was performed with treatment compliance as the outcome variable (behaviour) and the rest of HBM variables as predictors of the behaviour.

### Data Analysis

Data was analysed using SPSS software programme 17.0 version. The chi-square test was used to compare proportions. Multivariate analysis was performed using Linear Multiple Regression to obtain strongest predictor variable between variables of HBM. A p-value less than 0.05 was considered statistically significant.

### Ethical Approval

Muhimbili University of Health and Allied Sciences (MUHAS) approved the study (Ref. NO. MU/PGS/SAEC/Vol. VI). All participants gave their written consent to participate and were informed that they could terminate their participation at any time without incurring any cost.

## RESULTS

A total of 135 participants were included in the study. The mean age of participants was 56.3, ±13.1 years. Most of the participants 76 (56.3%) were females aged between 33 to 84 years. Majority of participants 82 (60.7%) were married. With respect to education level, most of the participants 74 (54.8%) had primary education. Regarding occupation, most of the participants 75 (55.6%) were unemployed (Table 1).

**TABLE 1:** Socio-Demographic Characteristics of Respondents N=135

Characteristics	Frequency (n)	Percentage (%)
**Age (years)**		
≤64	88	65.2
≥65	47	34.8
**Sex**		
Male	59	43.7
Female	79	56.3
**Marital status**		
Married	82	60.7
Separate	25	18.5
Widower	28	20.7
**Level of education**		
informal education	45	33.3
Primary education	74	54.8
Secondary education	16	11.9
**Occupation**		
Employed	60	44.4
Unemployed	75	55.6

### Factors Associated with Treatment Compliance

The association between socio-demographic factors and treatment compliance was explored. Treatment compliance was significantly associated with sex. Female participants had higher proportion of treatment compliance (63%) than males (46%) *(P=.044).* Participants with less than 64 years of age 50 (56.8%) had higher proportion of treatment compliance than participants with 65 and above years (53.2%) *p=.686.* Participants with secondary education had 10 (62.5) compliance compared to those with informal education 25 (55.6%). However, the difference was not statistically significant (Table 2).

**TABLE 2:** Relationship between Social Demographic Characteristics and Treatment Compliance

Characteristics	Treatment Compliance		*P-value*
	Non-compliance n=60 No (%)	Compliance n=75 No (%)	
**Age**			
≤64	38 (43.2)	50(56.8)	*.686*
≥65	22 (46.8)	25 (53.2)	
**Sex**			
Male	32 (54.2)	27 (45.8)	*.044*
Female	28 (36.8)	48 (63.2)	
**Marital status**			
Married	32 (39.0)	50 (61.0)	*.287*
Separate	13 (52.0)	12 (48.0)	
Widower	15 (53.6)	13 (46.4)	
**Education level**			
Informal education	20 (44.4)	25 (55.6)	*.277*
Primary education	30 (40.5)	44 (59.5)	
Secondary education	6 (37.5)	10 (62.5)	
**Occupation**			
Employed	27 (44.0)	33 (56.0)	*.908*
Unemployed	33 (45.0)	42 (55.0)	

### Association of Health Belief Model Variables with Treatment Compliance

Participants with high perceived severity were more compliant 39(57.4%) to hypertension treatment compared to those with low perceived severity who had only 36(53.7%) complaint to hypertensive treatment. However, the difference was not significant (*p=.672*). Those with high perceived susceptibility to hypertension complications were more compliant 45(66.2%) to hypertensive treatment compared to those with low perceived susceptibility, who had complaint of 30(57.4%) only and the difference was significant (*p=.012*). Participants with high perceived benefit of using antihypertensive treatment were more complaint 44(66.7%) to hypertensive treatment compared to those with low perceived benefit 31(44.9%) and the difference was significant *(p=.011).* Regarding perceived barrier, those with low perceived barrier were more complaint 57(77%) to treatment compared to those with high perceived barrier to hypertensive treatment (18(29.5), the difference was significant (*p=.000*). Regarding cues to action, those who had high remainders were more complaint 52(66.7%) to treatment compared to those with low remainders of using hypertensive treatment 23(40.4%) and difference was significant *(p=.002*) (Table 3).

**TABLE 3:** Association of Health Belief Model Constructs with Treatment Compliance

HBM variables	Treatment compliance		*P-value*
	Non-compliant n=60 n (%)	Compliant n=75 n (%)	
**Perceived Severity**			
Low	29 (42.6)	39 (57.4)	*.672*
High	31 (46.3)	36 (53.7)	
**Perceived Susceptibility**			
Low	37 (55.2)	30 (44.8)	*.012*
High	23 (33.8)	45 (66.2)	
**Perceived Benefit**			
Low	38 (55.1)	31 (44.9)	*.011*
High	22 (33.3)	44 (66.7)	
**Perceived Barrier**			
Low	17 (23.0)	57 (77.0)	*.000*
High	43 (70.5)	18 (29.5)	
**Cues to action**			
Low	34 (59.6)	23 (40.4)	*.002*
High	26 (23.3)	52 (66.7)	

### Health Belief Model Factors Predicting Treatment Compliance

Treatment compliance showed significant positive association with perceived benefit (r=0.27; *P=.001*) which means the higher the perceived benefit of using medicine the higher the treatment compliance. Treatment compliance showed significant negative association with perceived barrier to treatment (r=−0.53; *P=.000*), indicating that the higher the perceived barrier the lower the compliance. Treatment compliance showed positive association with cues to action (r=0.19; *P=.022*) which means that when people receive more reminders of the importance of adhering with treatment, they become more compliant.

Perceived severity of hypertension showed significant positive association with perceived susceptibility of getting hypertension complications (r=0.29; *p=.001*) indicating that the higher the perceived severity of hypertension disease the higher the perception of being vulnerable to hypertension complications. Perceived severity showed positive significant association with cues to action (r=0.2; *p=.019*) indicating that the higher the perception of severity of hypertension the higher the following of the cues to action (reminders). Perceived benefit of using medication showed significant negative association with perceived barrier (r=−0.45; *p=.000),* this meant that the higher the perception of benefit the lower the perception of barriers.

Also perceived benefit of using medication showed positive association with cues to action (r=0.32; *p=.000),* meaningthat the higher the perception of benefit the higher the perception of following reminders (Table 4).

**TABLE 4:** Correlation of Health Belief Model Variables with Treatment Compliance

Variables	1	2	3	4	5	6
1. Treatment compliance	–	0.104	0.141	0.274^**^	−0.528^**^	0.197^*^
2. Perceived severity		–	0.285^**^	0.090	−0.090	0.202^*^
3. Perceived susceptibility			–	−0.062	−0.061	−0.180^*^
4. Perceived benefit				–	−0.449^**^	0.323^**^
5. Perceived barrier					–	0.323^**^
6. Cues to action						–

* - p < 0.05,

** - p <0.001

### Health Belief Model Factors Associated with Treatment Compliance

Multivariate analysis indicated significant model fit for the data (F=11.19 and *P value=.000).* The amount of variance in treatment compliance that was explained by the predictors was 30.3% (R^2^=0.303) with perceived barrier being the strongest predictor of treatment compliance (β=−0.477; *P=.000).* A negative Beta Coefficient indicates a negative association between perceived barriers and treatment compliance. Other predictor variables were not statistically associated with treatment compliance (Table 5).

**TABLE 5:** Health Belief Model Factors Predicting Treatment Compliance

HBM variables	Beta	*P-value*
Perceived severity	0.092	*.238*
Perceived susceptibility	0.147	*.062*
Perceived Benefit	0.050	*.557*
Perceived barriers	0.477	*.000*
Cues to action	0.035	*.671*

R^2^ = 0.303; F = 11.19 (*P = .000*)

Behaviour = Compliance to treatment.

## DISCUSSION

This study explored factors affecting treatment compliance among hypertensive patients who were attending hypertension clinics in Dar es Salaam. This study was guided by Health Belief Model. The key findings in this study include positive association between age, sex, level of education, marital status and treatment compliance. We also found that the significant predictors of using HBM constructs were perceived susceptibility of being at risk of getting hypertension complications, perceived benefit of using medicine, perceived barrier to treatment and cues to action. After control of all factor variables among the construct of the HMB, the strongest predictor was perceived barrier.

The study revealed the percentage of treatment compliance to be 55.6% among study participants. Similarly, Imad et al reported that 55.9% of hypertension patients had good adherence to antihypertensive medication.^18^ The compliance rate of 55.6% in the present study was low compared to the findings in the study conducted by Okello et al in Uganda and Adidja et al in Cameroon in which the compliance rate was 85% and 67.7%, respectively.^19,20^

Our findings showed that compliance to antihypertensive was higher than that of the study conducted by Pallangyo et al in tertiary hospitals in Tanzania which reported that 25.3% were compliant to their hypertensive treatment.^21^ Study conducted by Bovet et al in Temeke Dar es Salaam, reported low (34%) adherence of patients to antihypertensive treatment.^17^ Similar to a study by Goweda and Shatla.^22^ The possible reason for the discrepancy observed in treatment compliance could be explained by the nature and type of hospitals included in our study. Our study considered hypertensive patients with no other complications while the study conducted by Pallangyo et al involved admitted patients with heart failure as a complication of uncontrolled hypertension.^21^.

The current study shows that; participants who were 64 and below years of age had higher level of treatment compliance compared to those with 65 and above. The results are comparable to those reported in the study conducted by Demoner et al in the city of Maringá and Choi et al in Korea which reported that young age group showed association with treatment compliance and older patients showed poor adherence to antihypertensive treatment.^23,24^ The possible explanation of these results may be that young people have higher income since they are able to work and thus can afford to buy medications when compared to older people. Another possible reason is that older people are more likely to have more than one disease due to aging which may have exposed them to using multiple drugs and in turn they become frustrated and, hence, stop taking drugs.^25^ Also cognitive and functional impairment in elderly patients increase their risk of poor drug compliance, thus they may require to have someone to remind, support and assist them in taking their drugs.^26^

Our study results revealed that female patients were more compliant to antihypertensive medication (63.2%) compared to male patients (*P=0.044*). Female patients have been reported to be better at adhering to antihypertensive treatment as compared to male patients.^27,28^

Impotence is the likelihood side effect which affects men's compliance with antihypertensive medications. This could be the reason why males showed low level of treatment compliance compared to females.^28,29^ The findings from the current study revealed that patients with secondary level of education had a higher level of treatment compliance to antihypertensive medications as compared to those with informal education (62.5 vs 37.5%). However, the difference was not significant. The probable reason could be that patients with high education level are likely to be more complaint to antihypertensive medications, due to the fact that having high education level make an individual to think critically about hypertension complications and also to have informed decision making about use of antihypertensive medications.^30^ This is similar to the study done by Saruna at el who reported that the level of education was significantly associated with treatment compliance.^30^ The same was reported by Yan et al in the study conducted in China.^28^ Also, Goweda and Shatla reported that patients with high level of education might be adherent to antihypertensive medication and life style modification since they understand the adverse effect of not being complaint to medication.^22^

The association between marital status and treatment compliance was revealed, in the current study, married participants were more compliant to medications (61%) when compared to single participants. Abbas et al also found that divorce was associated with poor adherence to antihypertensive medication (OR=2.14, 95% CI=1.31–5.48).^31^ Marriage might have a positive effect on compliance to medications. Partners might help each other in reminding each other the time for taking medications and also give moral support on the importance of treatment.

Perceived barrier was an important predictor in non-compliance to antihypertensive drugs and physical exercises.

This finding agreed with the study conducted by Yang et al in rural area of China which reported that adherence to antihypertensive medications is higher with less perceived barriers^32^ a scale based on the HBM, and the four-item Morisky Medication Adherence Scale. Results 745 hypertensive patients participated in the study (345 men, 400 women. The perceived barrier to antihypertensive medication was also reported by Obirikorang et al in the study conducted in Ghana.^33^ This is true according to the Health Belief Model: when a person perceives there is an obstacle of taking medication, he will not comply to his medication and exercise as supposed to and this will lead to complications and/or death.

Barriers of not complying with antihypertensive medication were determined. The reasons were; stopping medication due to cost of the medications^20,34^, fear of the side effect^35,36^, feeling well (asymptomatic)^37^, avoiding addiction to drugs^38,39^ and use of traditional medicine.^28^

## CONCLUSION

This study reported compliance to antihypertensive treatment of (55.6%) among study participants. Perceived barrier to treatment of hypertension was the strongest predictor among the constructs of HBM. Patients need advice, support and information from health professionals in order to understand the importance of using drugs as prescribed.

It is recommended that health care providers should be aware that hypertensive patients need to be educated on how to manage the disease and also be reminded continuously for better control of hypertension and improving the quality of their lives. This education and reminders should focus on the importance of complying to antihypertensive medications, physical exercises, diet and salt intake restriction. However, the HBM variables do not provide for advising patients the importance of treatment adherence, thus there is a need to use more than one theoretical model to provide adherence to antihypertensive advice to patients. Further studies should be conducted to assess why people have perceived barriers to treatment compliance.

### Limitation of the study

This study was conducted in Tanzania government regional hospitals in 3 Municipals of the Dar es Salaam region only. A cross sectional study design was used. Therefore, results cannot be generalised to all hypertension patients in Dar es Salaam because of the nature of the study design. Self-reporting of treatment compliance could introduce recall bias by either over reporting or under reporting depending on the patient's behaviour in the recent past. Based on the reason that this was a cross-sectional study, there is a possibility of recall bias in our study. In the current study HBM, remain descriptive and does not suggest the action for patients to change their behaviour. HBM should be used with other models so that patients can be advised to change their behaviours.

## References

[1] Bae SG, Kam S, Soo PK, Hong N, Kim K, Lee Y, et al. Factors related to intentional and unintentional medication nonadherence in elderly patients with hypertension in rural community. Dove Press J. 2016;1979–89. 10.2147/PPA.S114529 PMC5047725 27729776

[2] Zanello SB, Tadigotla V, Hurley J, Skog J, Stevens B, Calvillo E, et al. Inflammatory gene expression signatures in idiopathic intracranial hypertension: possible implications in microgravity-induced ICP elevation. npj Microgravity [Internet]. 2018 12 11;4(1):1. Available from: 10.1038/s41526-017-0036-6 29354685 PMC5764966

[3] Glanz K, Rimer B k., Viswanath K. Health and Health. 2002.

[4] Forouzanfar MH, Liu P, Roth GA, Ng M, Biryukov S, Marczak L, et al. Global Burden of Hypertension and Systolic Blood Pressure of at Least 110 to 115 mm Hg, 1990-2015. JAMA [Internet]. 2017 1 10;317(2):165–82. Available from: http://jama.jamanetwork.com/article.aspx?doi=10.1001/jama.2016.19043 28097354 10.1001/jama.2016.19043

[5] Twagirumukiza M, De Bacquer D, Kips JG, De Backer G, Vander R, Van Bortel LM. Current and projected prevalence of arterial hypertension in sub-Saharan Africa by sex, age and habitat : an estimate from population studies. 2008; 10.1097/HJH.0b013e328346995d 21540748

[6] Allanson ER, Muller M, Pattinson RC. Causes of perinatal mortality and associated maternal complications in a South African province: challenges in predicting poor outcomes. BMC Pregnancy Childbirth [Internet]. 2015 12 15;15(1):37. Available from: http://bmcpregnancychildbirth.biomedcentral.com/articles/10.1186/s12884-015-0472-9 25880128 10.1186/s12884-015-0472-9 PMC4339432

[7] Zack RM, Irema K, Kazonda P, Leyna GH, Liu E, Spiegelman D, et al. Determinants of High Blood Pressure and Barriers to Diagnosis andTreatment in Dar es Salaam, Tanzania. 2017;34(12):2353–64. 10.1097/HJH.0000000000001117 PMC5675737 27648720

[8] Seedat Y, Ali A, Ferdinand KC. Hypertension and cardiovascular disease in the sub-Saharan African context. 2018;6(6). 10.21037/atm.2018.06.45 PMC6123213 30211185

[9] Olga M, Prokop E, Migaj J, Grajek S. Is compliance with lifestyle modifications dependent on sociodemographic factors and awareness of HF symptoms? Impact of lifestyle changes on HF patients ' wellbeing. 2017;86(2):154–62.

[10] MoHCDGEC. Standard treatment Guideline & Essecial Medicine list in Tanzania Mainland. 2017.

[11] Mary Jande, Deogratias M Katabalo, Praveen Sravanam, Carol Marwa B, Madlan, Johanita Burger, Brian Godman, Margaret Oluka AM, Mwita. S. Patient Related-Beliefs and Adherence towards their Medications among the Adult Hypertensive Outpatients in Tanzania. 2017; 10.2217/cer-2016-0060 28485175

[12] Robinson N, Miller A, Wilbur J, Fogg L. Subjective Versus Objective Estimated Cardiovascular Disease Risk and Adherence to Physical Activity in African American Women. J Cardiovasc Nurs [Internet]. 2018 Mar;33(2):111–7. Available from: https://journals.lww.com/00005082-201803000-00005 28723836 10.1097/JCN.0000000000000437

[13] Oung AB, Kosirog E, Chavez B, Brunner J, Saseen JJ. Evaluation of medication adherence in chronic disease at a federally qualified health center. Ther Adv Chronic Dis. 2017;8(8—9):113–20. 28815008 10.1177/2040622317714966 PMC5546648

[14] Elmer A. A Health Belief Model-Based Childhood Immunization Intervention Within a Managed Care Organization. 2011;

[15] Rosenstock IM, Strecher VJ, Becker MH. Social Learning Theory and the Health Belief Model. Heal Educ Behav. 1988;15(2):175–83. 10.1177/109019818801500203 3378902

[16] National Bureau of Statistics. Tanzania 2015-16 Demographic Health Survey and Malaria Indicator Survey. Dar Es Salaam: Ministry of Finance; 2016. p. 24.

[17] Bovet P, Gervasoni J-P, Mkamba M, Balampama M, Lengeler C, Paccaud F. Low utilization of health care services following screening for hypertension in Dar es Salaam (Tanzania): a prospective population-based study. BMC Public Health [Internet]. 2008 12 16;8(1):407. Available from: https://bmcpublichealth.biomedcentral.com/articles/10.1186/1471-2458-8-407 19087300 10.1186/1471-2458-8-407 PMC2615777

[18] Alhaddad IA, Hamoui O, Hammoudeh A, Mallat S. Treatment adherence and quality of life in patients on antihypertensive medications in a Middle Eastern population: adherence. Vasc Health Risk Manag [Internet]. 2016 Oct;Volume 12:407–13. Available from: https://www.dovepress.com/treatment-adherence-and-quality-of-life-in-patients-on-antihypertensiv-peer-reviewed-article-VHRM 10.2147/VHRM.S105921 PMC5089866 27822055

[19] Okello S, Nasasira B, Muiru ANW, Muyingo A. Validity and Reliability of a Self-Reported Measure of Antihypertensive Medication Adherence in Uganda. Reboldi G. PLoS One [Internet]. 2016 7 1;11(7):e0158499. Available from: 10.1371/journal.pone.0158499 27367542 PMC4930194

[20] Adidja NM, Agbor VN, Aminde JA, Ngwasiri CA, Ngu KB, Aminde LN. Non-adherence to antihypertensive pharmacotherapy in Buea, Cameroon: a cross-sectional community-based study. BMC Cardiovasc Disord [Internet]. 2018 12 24;18(1):150. Available from: https://bmccardiovascdisord.biomedcentral.com/articles/10.1186/s12872-018-0888-z 30041606 10.1186/s12872-018-0888-z PMC6056997

[21] Pallangyo P, Millinga J, Bhalia S, Mkojera Z, Misidai N, Swai HJ, et al. Medication adherence and survival among hospitalized heart failure patients in a tertiary hospital in Tanzania: a prospective cohort study. BMC Res Notes [Internet]. 2020 12 21;13(1):89. Available from: 10.1186/s13104-020-04959-w 32085803 PMC7035643

[22] Goweda RA, Shatla MM. Adherence to Antihypertensive Medications: Rate and Predictors. Egypt Fam Med J [Internet]. 2020 5 1;4(1):95–109. Available from: https://efmj.journals.ekb.eg/article_90203.html

[23] Demoner MS, Ramos ER de P, Pereira ER. Factors associated with adherence to antihypertensive treatment in a primary care unit. Acta Paul Enferm. 2012;25(spe1):27–34.

[24] Sirasa F, Mitchell L, Silva R, Harris N. Factors influencing the food choices of urban Sri Lankan preschool children: Focus groups with parents and caregivers. Appetite. 2020;150:1–26. 10.1016/j.appet.2020.104649 32142823

[25] Bilal A, Riaz M, Shafiq Nulain, Ahmed M, Sheikh S, Rasheed S. Non-Compliance To Anti-Hypertensive Medication and Its Associated Factors Among Hypertensives. J Ayub Med Coll Abbottabad. 2015;27(1):158–63. 26182765

[26] Abegaz TM, Shehab A, Gebreyohannes EA, Bhagavathula AS, Elnour AA. Nonadherence to antihypertensive drugs a systematic review and meta-analysis. Medicine (United States). 2017. 10.1097/MD.0000000000005641 PMC5287944 28121920

[27] Tibebu A, Mengistu D, Bulto LN. Adherence to prescribed antihypertensive medications and associated factors for hypertensive patients attending chronic follow-up units of selected public hospitals in Addis Ababa, Ethiopia. Int J Health Sci (Qassim) [Internet]. 11(4):47–52. Available from: http://www.ncbi.nlm.nih.gov/pubmed/29085268%0Ahttp://www.pubmedcentral.nih.gov/articlerender.fcgi?artid=PMC5654188 PMC5654188 29085268

[28] Macquart de Terline D, Kane A, Kramoh KE, Ali Toure I, Mipinda JB, Diop IB, et al. Factors associated with poor adherence to medication among hypertensive patients in twelve low and middle income Sub-Saharan countries. Li Y. PLoS One [Internet]. 2019 7 10;14(7):e0219266. Available from: https://dx.plos.org/10.1371/journal.pone.0219266 31291293 10.1371/journal.pone.0219266 PMC6619761

[29] Amanda Almeida de Oliveira and Kenia Pedrosa Nunes. Hypertension and erectile dysfunction: breaking down the challenges. 2020;1–6. 10.1093/ajh/hpaa143 32866225

[30] Ghimire S, Pradhananga P, Baral BK, Shrestha N. Factors Associated With Health-Related Quality of Life among Hypertensive Patients in Kathmandu, Nepal. Front Cardiovasc Med [Internet]. 2017 11 6;4(November):1–8. Available from: http://journal.frontiersin.org/article/10.3389/fcvm.2017.00069/full 29164136 10.3389/fcvm.2017.00069 PMC5681715

[31] Abbas H, Kurdi M, Watfa M, Karam R. Adherence to treatment and evaluation of disease and therapy knowledge in Lebanese hypertensive patients. Patient Prefer Adherence. 2017;11:1949–56. 29238170 10.2147/PPA.S142453 PMC5713687

[32] Yang S, He C, Zhang X, Sun K, Wu S, Sun X, et al. Determinants of antihypertensive adherence among patients in Beijing: Application of the health belief model. Patient Educ Couns [Internet]. 2016;99(11):1894–900. Available from: 10.1016/j.pec.2016.06.014 27378081

[33] Obirikorang Y, Obirikorang C, Acheampong E, Odame Anto E, Gyamfi D, Philip Segbefia S, et al. Predictors of Noncompliance to Antihypertensive Therapy among Hypertensive Patients Ghana: Application of Health Belief Model. Int J Hypertens. 2018;2018. 10.1155/2018/4701097 PMC6029446 30018819

[34] Tajeu GS, Muntner P. Cost-related antihypertensive medication nonadherence: Action in the time of COVID-19 and beyond. Am J Hypertens. 2020;33(9):816–8. 32449903 10.1093/ajh/hpaa085 PMC7314165

[35] Khan M, Shah S, Hameed T. Barriers to and determinants of medication adherence among hypertensive patients attended National Health Service Hospital, Sunderland. J Pharm Bioallied Sci [Internet]. 2014;6(2):104. Available from: http://www.jpbsonline.org/text.asp?2014/6/2/104/129175 10.4103/0975-7406.129175 PMC3983739 24741278

[36] Hussein A, Awad MS, Mahmoud HEM. Patient adherence to antihypertensive medications in upper Egypt: a cross-sectional study. Egypt Hear J. 2020;72(1). 10.1186/s43044-020-00066-0 PMC7248145 32451726

[37] Osamor PE, Owumi BE. Factors associated with treatment compliance in hypertension in southwest Nigeria. J Heal Popul Nutr. 2011;29(6):619–28. 10.3329/jhpn.v29i6.9899 PMC3259725 22283036

[38] Leslie KH, McCowan C, Pell JP. Adherence to cardiovascular medication: a review of systematic reviews. J Public Health (Bangkok) [Internet]. 2019 3 1;41(1):e84–94. Available from: https://academic.oup.com/jpubhealth/article/41/1/e84/5020710 10.1093/pubmed/fdy088 PMC6459362 29850883

[39] Algabbani FM, Algabbani AM. Treatment adherence among patients with hypertension: findings from a cross-sectional study. Clin Hypertens. 2020;26(1):1–9. 32944283 10.1186/s40885-020-00151-1 PMC7491181

